# Peripheral Blood TCR Clonotype Diversity as a Biomarker for Colorectal Cancer

**DOI:** 10.3390/bioengineering12111215

**Published:** 2025-11-07

**Authors:** Gaochen Zhu, Tao Chen, Chen Ma, Kai Liu, Bihui Huang, Guan Yang

**Affiliations:** 1 Department of Infectious Diseases and Public Health, City University of Hong Kong, Kowloon, Hong Kong SAR 999077, China; gczhu2-c@my.cityu.edu.hk (G.Z.); kailiu@cityu.edu.hk (K.L.); 2 Department of General Surgery, Shanghai General Hospital, Shanghai Jiao Tong University School of Medicine, Shanghai 200080, China; tranco06@163.com; 3 Department of Computer Science, City University of Hong Kong, Kowloon, Hong Kong SAR 999077, China; chenma@cityu.edu.hk; 4 Scientific Research Center, The Seventh Affiliated Hospital, Sun Yat-sen University, Shenzhen 518107, China; huangbh7@mail.sysu.edu.cn

**Keywords:** TCR, machine learning, colorectal cancer, diagnosis

## Abstract

There exists an urgent need to improve colorectal cancer (CRC) diagnosis due to limitations in current diagnostic approaches. Systematic characterization of the human T cell receptor (TCR) repertoire, coupled with advanced computational methods, provides a promising opportunity to develop more accurate and less invasive diagnostic strategies for this major malignancy. The main objective of this work is to establish a TCR repertoire-based diagnostic model for CRC using machine learning algorithms and to identify the most significant features contributing to accurate diagnosis. Through comprehensive comparative analysis of several machine learning algorithms, our results demonstrated that the Transformer model exhibited superior performance capabilities. The trained model achieved an area under the receiver operating characteristic curve (AUC) of 0.973 in predicting disease status in the internal test set. Furthermore, TCR repertoire analysis from the independent test set demonstrated robust predictions with an AUC of 0.814. Notably, we identified a panel of 50 TCR repertoire features that showed a diagnostic AUC of 0.869 using these 50 TCR CDR3 sequences. Together, this TCR repertoire-based disease model demonstrates significant potential for clinical applications in CRC diagnosis and treatment response monitoring. Furthermore, similar diagnostic models could be established for other immune-related diseases based on disease-specific TCR repertoire data.

## 1. Introduction

Colorectal cancer (CRC) ranks among the top three most commonly diagnosed cancers globally and continues to be a significant contributor to cancer-related deaths [1]. Despite decades of cancer research, the fundamental mechanisms responsible for the initiation and progression of this ubiquitous disease have not been fully elucidated [2]. Early diagnosis of CRC is crucial for ensuring appropriate treatment, as the cancer can metastasize to other organs, particularly the liver and lungs, leading to secondary tumors and associated complications [3]. However, current diagnostic approaches such as colonoscopy, fecal occult blood test, and imaging technology have notable limitations, such as the risk of perforation, false-positive results, and missed non-bleeding lesions [4]. Therefore, there is an urgent need to explore and develop effective diagnostic approaches for CRC.

T lymphocytes play a crucial role in the immune response against tumors by recognizing tumor-associated antigens through T cell receptors (TCRs). TCR specificity and diversity are derived from the variable complementarity determining region 3 (CDR3) and the random rearrangement and mutations of Variable (V), Diversity (D), and Joining (J) regions. Numerous studies have highlighted the significance of TCR and CDR3 diversity in cancer diagnosis, therapy, and prognosis [5,6,7]. Thus, peripheral blood TCR repertoire profiling is emerging as a promising diagnostic tool for CRC, reflecting dynamic changes in the TCR repertoire that could serve as biomarkers for monitoring immunomodulatory processes.

Machine learning (ML), an advanced artificial intelligence technique for analyzing complex data, has proven successful in diagnosing and predicting various diseases, including cardiovascular diseases, cancer, and other immune-related disorders by analyzing the huge amount of microbiota data [8,9]. However, gut microbiota-based ML methods for cancer diagnosis have shown limitations, including insufficient biological evidence supporting microbiome-phenotype associations [10] and a high rate of misdiagnosis when applied to new populations [11]. In contrast, TCR-based ML methods can identify and predict tumor-associated antigens with high specificity, offering promising prospects for cancer diagnosis. In addition, this approach leverages the natural surveillance capabilities of the immune system against malignancies [12]. Therefore, investigating the diagnostic value of TCR repertoire-based ML in CRC represents a valuable research endeavor.

In recent years, several ML methods have been proposed to improve cancer prediction and diagnosis using TCR repertoire data. For instance, Ostmeyer et al. decomposed TCR sequences into 4-mer motifs and developed a logistic regression-based method for classifying tumor tissues from normal tissues [13]. Similarly, Beshnova et al. developed a convolutional neural network model called DeepCAT for cancer-associated TCR detection, which can further differentiate healthy individuals from cancer patients [14]. Despite these advancements, existing methods face challenges such as the need for large-scale labeled datasets, the complexity of deep learning models leading to interpretability issues, and potential overfitting due to high-dimensional TCR data [15,16]. Therefore, it is essential to develop alternative approaches that balance model complexity and interpretability while effectively utilizing TCR repertoire information for CRC diagnosis.

In this study, we propose a ML approach combined with feature selection techniques to construct a diagnostic model that addresses these challenges. Here, we construct a diagnosis model that utilizes peripheral blood TCR repertoire profiling to diagnose CRC and identify key biomarkers for CRC diagnosis. The findings are validated using publicly available TCR repertoire datasets, further strengthening the potential of this approach in cancer diagnostics. This approach could afford rapid and accurate classification from minimally invasive peripheral blood assays, providing biologically interpretable features and strong cross-cohort generalizability, thereby enabling safe, scalable deployment with reduced overfitting.

## 2. Materials and Methods

### 2.1. Data Collection

The TCR repertoire data were retrieved from the Sequence Read Archive database, available on the National Center for Biotechnology Information platform, and the Genome Sequence Archive in the National Genomics Data Center. A total of 220 CRC samples were collected from the following BioProjects: PRJNA754274 [17], which included 83 samples from 16 patients, with each patient having one or more samples sequenced; PRJCA009632 [18], 107 samples from 107 patients; and PRJNA1049886 [19], 30 samples from 30 patients. The 278 non-CRC samples were from the following BioProjects: PRJNA754274 [17], 20 samples from 20 healthy controls; PRJNA930724 [20], 22 samples from 22 healthy controls; PRJEB40492, 46 samples from 46 healthy controls; PRJEB50045 [21], 183 samples from 99 healthy individuals; and PRJNA821039 [22], 7 samples from 7 individuals. Apart from the four samples within BioProject PRJNA821039 that are from GATA2-deficient individuals, all remaining non-CRC samples are from healthy controls. All CRC patients enrolled in this study were recruited from China. The control group consisted of individuals from China and several European countries, including the United Kingdom, Germany, and Switzerland. A total of 347 subjects were recruited from multiple institutions, including the European Bioinformatics Institute, the Third Affiliated Hospital of Shandong First Medical University China, etc. The demographics of subjects recruited in this study are shown in Table S1. The discovery cohort included 123 CRC patients and 187 healthy individuals, which was split into training and internal test sets at a 70/30 ratio. The independent validation cohort, including 30 CRC patients and 7 non-CRC individuals, was used for external verification.

### 2.2. Bioinformatics and Statistical Analyses for the TCR Repertoire

The raw sequence data underwent quality filtering using Trimmomatic (V.39) to eliminate adaptor sequences and low-quality reads (quality score < 30). MiXCR (v.4.5.0) was employed to align the sequencing data to the reference sequences for the V, D, J, and Constant (C) gene segments of the TCR [23]. Following alignment, the data were assembled to determine the specific gene region sequences on the TCRβ, particularly the CDR3. Batch effect adjustment was performed using ComBat-seq, a tool for untransformed raw count data [24], to harmonize TRBV and TRBJ gene usage from different datasets generated by different labs. This methodology has been well applied in RNA-seq analyses, and has shown significant effects in reducing batch effects [25,26]. Additionally, a normalization strategy, where the clone counts from one cohort were used as a baseline, and the clone counts in other cohorts were scaled proportionally, was applied. This approach has been successfully utilized in previous TCR repertoire and single-cell RNA-seq studies to ensure comparability across samples [27,28]. Shannon diversity is computed as

(1) $$H=-\sum_{i=1}^{N}p_{i}\log_{2}p_{i},$$

where *p_i_* is the frequency of sequence *i* in the repertoire and *N* is the total number of unique sequences.

(2) $$\mathrm{Clonality}=1-\frac{H}{\log_{2}\left(N\right)}$$

All statistical analyses were conducted using R software (v.4.3.1). All the comparisons between the healthy control (HC) and CRC groups were performed using the Mann–Whitney U test with false discovery rate (FDR) correction for multiple testing. Statistical significance was set at three levels: * *p* < 0.05, ** *p* < 0.01, and *** *p* < 0.001.

Data visualization was performed using R software. Additionally, GraphPad Prism software (v9.0.0) was used for graphical visualizations.

### 2.3. Supervised Machine Learning

**Figure 1:**
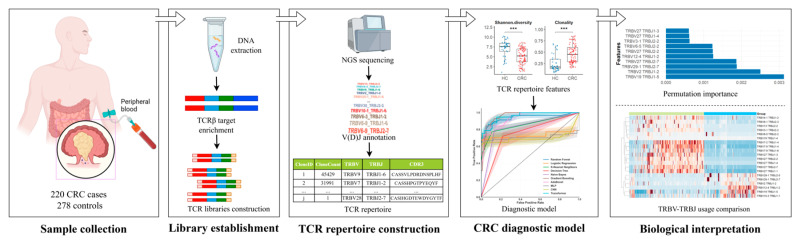
Schematic illustration of the pipeline in TCR repertoire analysis. Framework for sample collection, library establishment, TCR repertoire construction, CRC diagnostic model, and biological interpretation. All comparisons were performed using FDR-corrected Mann–Whitney U test, *** *p* < 0.001.

The data processing and analysis workflow is presented in Figure 1. In the classification of CRC versus non-CRC, seven different supervised ML algorithms, which were Random Forest (RF), Logistic Regression (LR), K-Nearest Neighbors (KNN), Decision Tree (DT), Naïve Bayes, Gradient Boosting, and Adaptive Boosting (AdaBoost), and three different deep learning (DL) algorithms, which were multiple layer perceptron (MLP), convolutional neural network (CNN), and Transformer, were trained with the features of the TCR repertoire. CRC samples are labeled as positive and non-CRC samples are labeled as negative.

Among these algorithms, RF is a powerful ensemble learning method employed for classification tasks [29]. It builds multiple decision trees during training, with each tree constructed on a data sample extracted from a training set. The output of RF is the class selected from the most trees for classification tasks. LR is a statistical method used for binary classification, modeling the relationship between dependent and independent variables to predict the probability of occurrence within a binary outcome [30]. KNN is a non-parametric method used for classification and regression, identifying the majority class of k-nearest neighbors to the query point in the feature space [31]. DT is a powerful and widely used classification model in data mining and machine learning, which efficiently partitions data by recursively testing numeric features against threshold values [32]. Naïve Bayes classification is a probabilistic model based on Bayes’ theorem, assuming independence among predictors, which simplifies the computation of conditional probabilities [33]. Gradient Boosting is a machine learning technique that constructs an additive model in a forward stage-wise fashion. It iteratively trains a series of weak learners, typically decision trees, where each subsequent model focuses on fitting the residual errors of the preceding model [34]. AdaBoost is an iterative algorithm that combines multiple weak classifiers into a strong classifier by adjusting the weights of training samples in each training, focusing on samples misclassified in the previous round, thereby improving the classification performance of the model [35]. MLP is a feed-forward neural network with multiple fully connected layers and nonlinear activations, capable of modeling complex, nonlinear relationships in high-dimensional data [36]. CNN is a specialized neural network architecture that employs convolutional layers to automatically extract hierarchical features from sequential or structured data, followed by pooling into fully connected layers for classification [37]. Transformer is an attention-based model that leverages self-attention mechanisms to process sequential data in parallel, excelling in capturing long-range dependencies without reliance on recurrence or convolution [38,39]. The Transformer algorithm’s robustness against overfitting in high-dimensional spaces, its ability to model positional encodings for sequence data, and its capacity to provide interpretable attention weights make it particularly suitable for TCR repertoire analysis.

We analyzed the CDR3 amino acid sequences from each sample, using their normalized frequencies as quantitative features. All unique CDR3 sequences across samples formed the feature set, with the top 1000 most frequent clones selected globally and further reduced to 500 via ANOVA F-value feature selection. Each sample was transformed into a feature vector where each element corresponds to the fraction of a specific CDR3 sequence. Missing values (i.e., sequences not present in a sample) were set to zero. The final input was a feature matrix X (n × m), where n is the number of samples, and m is the number of selected unique CDR3 sequences identified across all samples. The label y was assigned as ‘CRC’ for colorectal cancer samples and ‘HC’ for healthy controls.

Categorical labels were encoded into binary values (1 for ‘CRC’ and 0 for ‘HC’) using scikit-learn’s LabelEncoder. The discovery cohort was then split into training and internal test sets using a stratified 70/30 split to maintain the proportion of classes in both sets which is essential to ensure balanced representation of both classes in the training and testing datasets.

We employed stratified k-fold cross-validation with five folds (n_splits = 5), ensuring that each fold had a similar class distribution. For each algorithm, hyperparameter tuning was performed to optimize model performance. We defined parameter grids and utilized scikit-learn’s GridSearchCV for exhaustive search over specified parameter values. The models and their associated hyperparameters are as follows:

Random Forest: Number of estimators (n_estimators), maximum depth (max_depth), maximum features (max_features), and class weights (class_weight).

Logistic Regression: Regularization strength (C), penalty (penalty), and solver (solver).

K-Nearest Neighbors: Number of neighbors (n_neighbors) and weighting function (weights).

Naïve Bayes: Since the Gaussian Naïve Bayes classifier does not have hyperparameters that require tuning, it was used with default settings.

Decision Tree: Maximum depth (max_depth), minimum samples required to split (min_samples_split), and class weights (class_weight).

Gradient Boosting: Number of estimators (n_estimators), learning rate (learning_rate), and maximum depth (max_depth).

AdaBoost: Number of estimators (n_estimators) and learning rate (learning_rate).

Multilayer Perceptron: Hidden layer sizes (hidden_sizes), dropout rate (dropout), learning rate (learning_rate), number of epochs (epochs), and batch size (batch_size).

Convolutional Neural Network: Number of convolutional layers (conv_layers), filters per layer (filters), kernel size (kernel_size), pooling type (pooling), dropout rate (dropout), learning rate (learning_rate), number of epochs (epochs), and batch size (batch_size).

Transformer: Embedding dimension (d_model), number of attention heads (nhead), number of encoder layers (num_layers), dropout rate (dropout), learning rate (learning_rate), number of epochs (epochs), and batch size (batch_size).

More detailed model parameters were depicted in Table S2. Models were trained on the training folds and validated on the validation fold within the cross-validation framework. The hyperparameters yielding the highest mean AUC score during cross-validation were selected for each model. Accuracy, area under the receiver operating characteristic curve (AUC), recall, and F1-score were employed to measure the model performance. These are well-known evaluation metrics and have been used in previous bioinformatics studies [40,41].

After hyperparameter tuning and model training, we evaluated the models on the internal test set. We computed the mean and standard deviation of accuracy and AUC across the five folds to assess the stability and generalization capability of each model. The best-performing model from the cross-validation (based on AUC) was evaluated on the held-out test set to assess its predictive performance on unseen data. For each model, we generated the receiver operating characteristic (ROC) curves by plotting the true positive rate (sensitivity) against the false positive rate (1—specificity) at various threshold settings. Finally, we employed the optimal model to verify the AUC on the independent external validation cohort.

The analysis was conducted using Python (v.3.6.13) with the following libraries: pandas (v.1.1.5), NumPy (v.1.19.2), scikit-learn (v.0.24.2), and Matplotlib (v.3.3.4). Additionally, torch (v.2.5.1) was used for implementing deep learning models.

### 2.4. Feature Importance Measures

To assess the impact of each feature on the classifier’s predictive performance, we employed permutation importance within a cross-validation framework. This model-agnostic method measures the decrease in model performance when a single feature’s values are randomly shuffled, providing an unbiased estimation of feature importance suitable for high-dimensional datasets.

We used a five-fold stratified cross-validation to ensure that each fold had an approximately equal proportion of CRC and healthy samples, which is crucial to prevent class imbalance from affecting the model evaluation and the permutation importance estimates. For each feature in the validation set, we performed random shuffling of its values to disrupt any relationship with the target variable. The decrease in model performance was computed by comparing the model’s performance on the original validation data versus the permuted data. The importance score for each feature was calculated as the mean decrease in performance over 5 shuffling iterations (n_repeats = 5), balancing reliability and computational cost. The importance scores from each fold were accumulated and averaged across all folds to obtain a stable estimate of each feature’s importance.

## 3. Results

### 3.1. Comparison of TCR Repertoires Between CRC Patients and Healthy Controls

**Figure 2:**
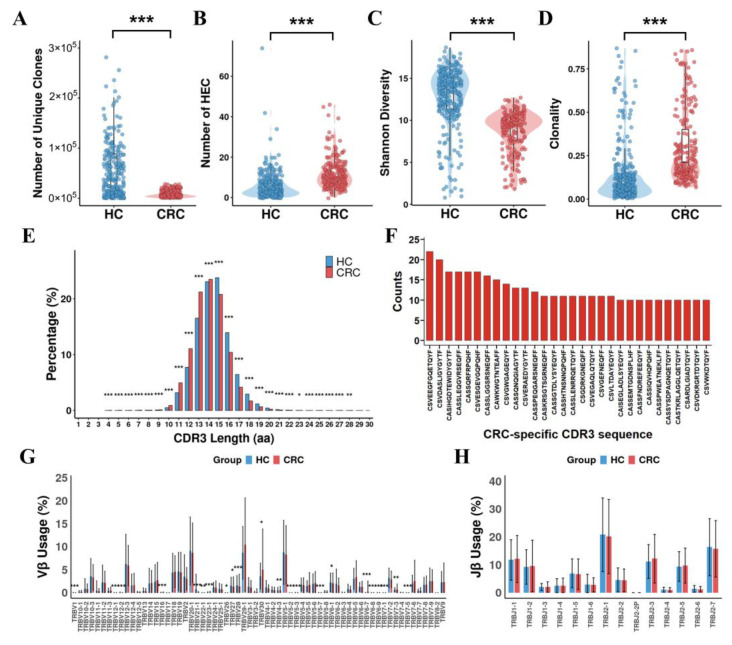
Comprehensive analysis of TCR repertoire characteristics and differential Vβ and Jβ gene usage in CRC patients and healthy controls (HC). (**A**) Comparison of unique clone number between HC and CRC patients. Each data point represents an individual sample. (**B**) Comparison of HEC number distribution between HC and CRC patients. (**C**) TCR repertoire Shannon Diversity comparison. (**D**) TCR repertoire Clonality comparison. (**E**) TCRβ amino acid sequence length distribution comparison. (**F**) CRC-specific sequence diagram. The top 30 CRC-specific CDR3 sequences were shown in the histogram. (**G**) Vβ gene usage comparison. (**H**) Jβ gene usage comparison. All comparisons were performed using FDR-corrected Mann–Whitney U test, * *p* < 0.05, ** *p* < 0.01, *** *p* < 0.001.

We reanalyzed the TCRβ repertoires in the peripheral blood of 187 healthy individuals and 123 CRC patients in the training and test sets [17,20]. Each sample exhibited distinct features in TCR diversity. Healthy individuals exhibited significantly higher number of unique TCR clones compared to CRC patients (Figure 2A). TCR clones with a frequency above 0.5% of total reads in a sample were defined as high-expansion clone (HEC) [18]. The comparison of HEC numbers between the CRC and HC groups showed a significantly higher HEC ratio in the CRC group (Figure 2B). Additionally, healthy donors had relatively high TCR diversity and low TCR clonality (Figure 2C,D). The length distributions of TCRβ CDR3 in healthy individuals and CRC patients are different (Figure 2E). CDR3 amino acid lengths of shorter than or equal to 14 were more frequent in CRC patients than in healthy individuals while CDR3 amino acid lengths of longer than 14 were more frequent in healthy individuals. Subsequently, we eliminated the sequences that appeared in the healthy group and ranked the remaining CRC-specific CDR3 sequences according to the occurrence. The top 30 of CRC-specific CDR3 sequences are shown in the histogram (Figure 2F). Furthermore, significantly higher usages of TRBV1, TRBV4-3, TRBV5-2, TRBV5-3, TRBV5-7, TRBV6-1, TRBV6-8, TRBV6-9, TRBV7-1, TRBV7-3, TRBV7-5, TRBV12-1, TRBV12-2, TRBV21-1, and TRBV22-1 were observed in healthy donors compared to CRC patients among the functional human Vβ genes. Conversely, significantly higher usages of TRBV6-7, TRBV16, TRBV23-1, and TRBV30 were observed in CRC patients compared to healthy donors (Figure 2G, FDR-corrected Mann–Whitney U test, * *p* < 0.05, ** *p* < 0.01, *** *p* < 0.001). However, among the functional Jβ genes, no significant differences were observed between CRC patients and healthy individuals. (Figure 2H, FDR- corrected Mann–Whitney U test). These results demonstrate the consistency of the TCR repertoire across different CRC datasets and further substantiate the potential of the TCR repertoire as a biomarker for diagnosing CRC.

### 3.2. Performance of TCR Repertoire-Based ML Models for CRC Diagnosis

**Figure 3:**
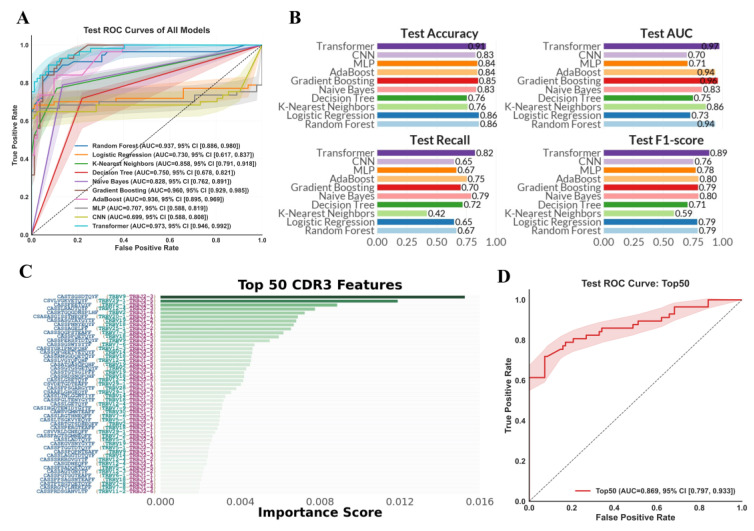
Machine learning models for diagnosing CRC. (**A**) The ROC curve for the internal test set across 7 machine learning (ML) and 3 deep learning (DL) methods. (**B**) Comparative analysis of the internal test set accuracy, AUC, recall, and F1-score across 7 ML and 3 DL methods. (**C**) The top 50 feature importances determined by permutation importance for the Transformer model. (**D**) The ROC curve of the Transformer model using top 50 features for the internal test set.

Given that TCR diversity is primarily determined by CDR3, our study focused on the usage of CDR3 sequences. We constructed CRC diagnostic models using CDR3 amino acid sequences and their corresponding fractions as the input features. To evaluate the effectiveness of various ML and DL algorithms in diagnosing CRC based on TCR repertoire data, we assessed the AUC of ten different models on the internal test set: RF, LR, KNN, DT, Naive Bayes, Gradient Boosting, AdaBoost, MLP, CNN, and Transformer. For each algorithm, we searched across the parameter space specified in Table S2 to identify the optimal hyperparameter configuration. We then trained the model using the configuration that achieved the best performance under five-fold cross-validation and evaluated it on the test set. As illustrated in Figure 3A, the ROC curves obtained from the internal test set demonstrate that the Transformer model achieved the highest overall performance, with an AUC value of 0.973.

We further validated the accuracy, recall, and F1-score of these models on the internal test set (Figure 3B). The results indicate that the Transformer model maintained superior performance, achieving an accuracy of 0.91, recall of 0.82, and F1-score of 0.89. In contrast to another artificial intelligence-assisted technique for identifying CRC through blood tests [42], which consists of a binary SVM classifier at the first level and a one-class SVM classifier at the second level (achieving 60% sensitivity and 79% specificity), and the use of Red Cell Distribution Width [43], which demonstrated a sensitivity of 84% and a specificity of 88% for right-sided colon cancer, our model demonstrates superior performance.

In summary, these results demonstrate that TCR repertoire profiling, when analyzed through ML models, can serve as a highly accurate and robust diagnostic marker for CRC, presenting significant potential for clinical applications in early detection.

### 3.3. Identification of Key TCR Repertoire Biomarkers

In our analysis, to identify significant TCR repertoire biomarkers for CRC diagnosis, we employed a ML model based on Transformer enhanced by permutation importance to quantify the impact of individual TCR features on disease classification. Figure 3C and Table S3 showed the top 50 TCR features ranked by their permutation importance scores. Among these, CASTSGSDTQYF (TRBV9/TRBJ2-3) showed the highest importance score, indicating its critical role in differentiating CRC from healthy controls. Additionally, these top 50 features yielded an AUC of 0.869 and an accuracy of 0.8273 for the diagnosis of CRC (Figure 3D).

We retrained the model using the top 30, top 40, and top 50 features and subsequently plotted the ROC curves on the internal test set (Figure S1A). We also compared the AUC on the external test set across these three models (Figure S1B). We found that the model trained with the top 50 features achieved the highest AUC on the internal and external test set, recording values of 0.869 and 0.752, respectively. This result underscores the importance of the 50 features identified for the model.

### 3.4. Validation of TCR Repertoire-Based Models for CRC Diagnosis in Independent Cohorts

**Figure 4:**
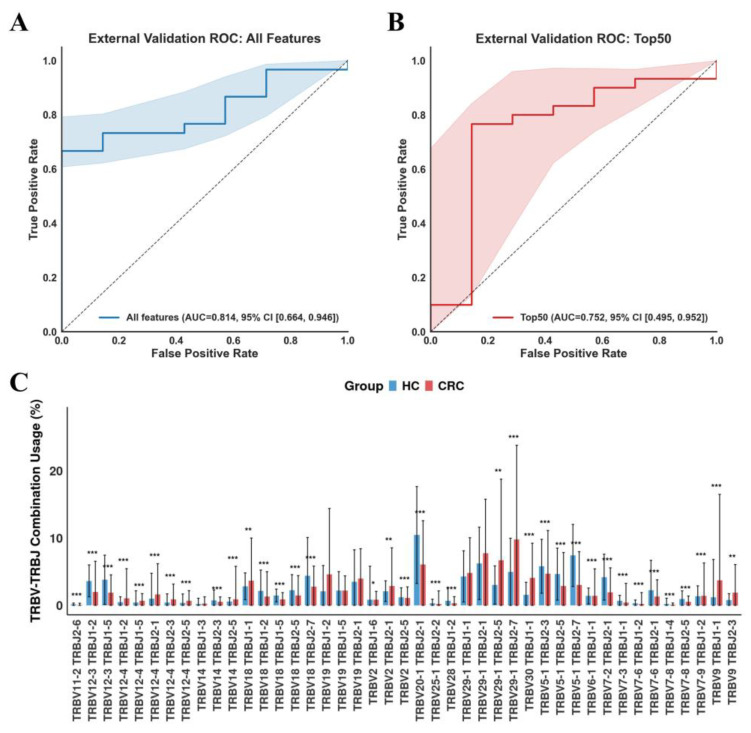
Validation of Transformer models on external validation datasets. ROC curves of CRC diagnostic model using all CDR3 features (**A**) and top 50 features (**B**) on external validation datasets. (**C**) Top 50 TRBV-TRBJ gene combination usage comparison. FDR-corrected Mann–Whitney U test, * *p* < 0.05, ** *p* < 0.01, *** *p* < 0.001.

To externally validate the diagnostic value and mitigate the risk of over-optimistic reporting of diagnostic accuracy, we assessed the Transformer model using all features and Top 50 features in the independent test sets. The Transformer model using all features showed an AUC of 0.814 (Figure 4A) and an accuracy of 0.8108, while the 50-marker model reached an AUC of 0.752 (Figure 4B) and an accuracy of 0.7568.

To further investigate the patterns of these key TCR features across individual samples, we employed an FDR-corrected Mann–Whitney U test to compare the CDR3-derived TRBV-TRBJ combination usage, which was normalized by Combat-seq. In total, 37 of the top 50 TRBV-TRBJ combinations showed a significant difference between healthy individuals and CRC patients (Figure 4C), with FDR-corrected *p* < 0.05, indicating their potential association with CRC status. This analysis not only underscores the utility of TCR repertoire analysis for identifying potential diagnostic markers but also suggests that certain combinations of TCR gene segments may be intimately involved in the immune response to CRC.

These findings provide a valuable foundation for further investigations into the TCR repertoire in CRC and may contribute to the development of novel diagnostic and therapeutic strategies targeting specific TCR interactions.

## 4. Discussion

### 4.1. Peripheral Blood TCR Repertoire Profiling Enables Accurate, Non-Invasive CRC Diagnosis

In this study, we demonstrate that peripheral blood TCR repertoire profiling, integrated with machine learning, offers a highly accurate and non-invasive approach for CRC diagnosis. Our data suggest alterations in TCR diversity and clonality in the peripheral blood of CRC patients compared with healthy individuals, highlighting the potential of TCR signatures as biomarkers. Notably, we successfully identified a panel of 50 key TCR biomarkers that differentiate CRC patients from healthy individuals, with CASTSGSDTQYF (TRBV9/TRBJ2-3) emerging as the most discriminative feature based on permutation importance. Indeed, increasing evidence supports the use of deep sequencing-based TCR repertoires as biomarkers for immune response in cancer patients [44]. Emerging techniques utilizing TCR gene sequencing offer novel approaches to evaluate lymphocytic infiltration, providing valuable insights into both clonality and abundance [45]. High clonality suggests the presence of a few dominant clones, while low clonality indicates the existence of multiple clones with similar abundance. Our observation of elevated clonality in CRC patients (Figure 2D) suggests oligoclonal expansions potentially elicited by tumor neoantigens, a phenomenon observed across various malignancies [46,47]. The specific TRBV-TRBJ patterns we identified, such as TRBV18/TRBJ2-7, thus represent a discriminatory immune signature for CRC that warrants further mechanistic exploration.

### 4.2. Key TCR Repertoire Features Underpin Robust Performance

We found that CRC patients exhibit significant differences in the usage of specific Vβ gene segments in the TCRs compared to healthy individuals. Certain Vβ genes, such as TRBV6-7, TRBV16, TRBV23-1, and TRBV30 were overrepresented in CRC patients (Figure 2G). These findings suggest that certain TCR gene segment usages are associated with CRC, potentially reflecting an adaptive immune response to tumor-associated antigens. Similar patterns have been observed in other cancers, where particular TCR clonotypes are enriched in affected patients [48]. These data underscore the utility of TCR repertoire analysis in identifying potential diagnostic markers and providing insights into the immune mechanisms involved in CRC.

Our data further demonstrate the feasibility of utilizing the TCR repertoire-based model for CRC diagnosis. Notably, our innovative focus on CDR3 sequences features, rather than solely on TRBV-TRBJ gene combination, has led to enhanced model performance compared to previous methods. By leveraging these specific TCR clones, we have improved the predictive accuracy for CRC diagnosis, highlighting the potential of these features as robust disease biomarkers. Compared to established CRC diagnostics like colonoscopy (sensitivity ~95% for advanced lesions but <70% for early polyps [4]), our TCR-based model offers a non-invasive alternative with potential for early-stage detection via immune profiling, as evidenced by CDR3 sequences and differential TRBV-TRBJ usages in peripheral blood from datasets including pre-treatment CRC samples [17]. Future prospective studies in early-stage cohorts will further validate its clinical merit. Incorporating the top 50 TCR features also yielded high diagnostic accuracy, suggesting that a refined biomarker panel could streamline clinical implementation. Importantly, several of these features show overlap with signatures in other cancers. For instance, the TRBV18/TRBJ2-7 combination corresponding to CASSPNNYEQYF in our panel has also been reported as a top contributor to CRC prediction models in lymph node metastasis studies [19]. Similarly, TRBV19/TRBJ1-2 combinations, which correspond to highly expanded clones such as CASKGVSNYGYTF and CASSASGTAYGYTF in our dataset, were among the most frequent in lymph nodes and peripheral blood mononuclear cells of papillary thyroid carcinoma patients [49]. Additionally, the TRBV7-2/TRBJ2-1 combination, which underlies CASSFAGTSGMNEQFF in our results, represents one of the most abundant segments identified in clear cell renal cell carcinoma [50]. These cross-cancer associations imply shared immunogenic motifs or convergent evolution in anti-tumor immunity, with implications for pan-cancer biomarkers and personalized therapies targeting conserved TCR-antigen interactions.

### 4.3. Clinical Implications, Limitations, and Future Directions

Although our findings advance TCR-based diagnostics, several limitations should be acknowledged. Firstly, the sample population primarily consisted of individuals from Asia (China) and Europe (the United Kingdom, Germany, and Switzerland). This geographic limitation may affect the generalizability of our findings to other populations with different genetic backgrounds and environmental exposures. Future studies should include more diverse populations to validate and extend our findings. Additionally, while next-generation sequencing costs have decreased to approximately $50 per sample, enabling scalability via cloud-based machine learning pipelines, integration into routine clinical workflows requires standardized protocols and automated bioinformatic tools to enhance efficiency and accessibility. Finally, regulatory approval for TCR profiling as a diagnostic assay remains a key challenge, necessitating prospective clinical trials to establish sensitivity, specificity, and cost-effectiveness in real-world settings. Future research should focus on multi-center collaborations spanning underrepresented regions, the development of universally accepted workflow standards, and the design of prospective validation studies in diverse healthcare contexts. Addressing these limitations through future research will be critical to translating this approach into clinical practice.

In conclusion, our data indicated that TCR diversity and clonality in peripheral blood could be used for CRC diagnosis. Our model can also be extended for the prediction and diagnosis of other immune-mediated diseases by analyzing disease-specific TCR repertoire and implementing our ML approach based on CDR3 sequences.

## 5. Patents

A US provisional patent (No. 63/625,326, Title: TCR repertoire-based machine learning for colorectal cancer diagnosis) has been filed based on this work. A China invention patent has also been filed based on this work (No. 202510107763.4, Title: TCR repertoire-based machine learning for colorectal cancer diagnosis).

## Data Availability

The original data presented in the study are openly available in the National Center for Biotechnology Information platform [PRJNA754274, PRJCA009632, PRJEB40492, PRJEB50045, PRJNA821039].

## Supplementary Materials

The following supporting information can be downloaded at: https://www.mdpi.com/article/10.3390/bioengineering12111215/s1, Figure S1: Performance of Transformer models employing different numbers of features in the test cohort; Table S1: Demographics of subjects recruited in this study; Table S2: Parameters employed to construct different ML models; Table S3: Top 50 TCR features ranked by permutation importance.
