# Supplementary material for: Peripheral Blood TCR Clonotype Diversity as a Biomarker for Colorectal Cancer
Source: Bioengineering (Basel). 2025 Nov 7;12(11):1215. doi: 10.3390/bioengineering12111215 (PMC12650616; doi:10.3390/bioengineering12111215)
Supplement: Supplementary file 1 [file bioengineering-12-01215-s001.zip › bioengineering-3958394-supplementary.pdf]

Table S1. Demographics of subjects recruited in this study.

Cohorts	Source	Group	n	Sex	Age (ave ± SD)	Geo	Race
Discovery	Chen. Y.-T., et al., 2022 [17]	CRC	16	Female, 34.5%	62.38 ± 12.62	China	Asian
		HC	20	Female, 55%	62.6 ± 10.48		
	Cao, Y., et al., 2023 [18]	CRC	107	Female, 43.9%	63.28 ± 12.95		
	Malik, A., et al., 2023 [20]	HC	22	Female, 50%	54 ± N/A	UK	N/A
	Rosati, E., et al., 2022 [21]	HC	99	Female, 59%	48 ± 13	Germany	Caucasian
	European Bioinformatics Institute	HC	46	Female, 63%	35.43 ± 14.29	N/A	N/A
Validation	The Third Affiliated Hospital of Shandong First Medical University	CRC	30	Female, 46.7%	63.23 ± 11.94	China	Asian
	Von, N., et al., 2023 [22]	GATA2 deficient	4	Female, 75%	17 ± 6.13	Swiss	Caucasian
		HC	3	Female, 33.3%	31.67 ± 16.74		

Notes: CRC, colorectal cancer; HC, healthy control; ave, average; SD, standard deviation; Geo, Geolocation.

Table S2. Parameters employed to construct different ML models.

Parameter	RF	LR	KNN	DT	NBM	GBDT	AdaBoost	MLP	CNN	Transformer
**n_estimators**	100, 200, 300	/	/	/	/	100, 200	50, 100, 200	/	/	/
**max_depth**	None, 10, 20, 30	/	/	None, 5, 10	/	3, 5, 10	/	/	/	num_layers: 1, 2, 3
**max_features**	'auto', 'sqrt', 'log2'	/	/	/	/	/	/	/	/	d_model: 32, 64, 128
**class_weight**	None, 'balanced'	/	/	None, 'balanced'	/	/	/	/	/	/

**C**	/	0.01, 0.1, 1, 10	/	/	/	/	/	/	/	/
**penalty**	/	'l2'	/	/	/	/	/	/	/	/
**solver**	/	'lbfgs'	/	/	/	/	/	/	/	/
**n_neighbors**	/	/	3, 5, 7	/	/	/	/	/	/	/
**weights**	/	/	'uniform', 'distance'	/	/	/	/	/	/	/
**min_samples _split**	/	/	/	2, 5, 10	/	/	/	/	/	/
**learning_rate**	/	/	/	/	/	0.01, 0.1, 1	0.01, 0.1, 1	0.001, 0.0001	0.001, 0.0001	0.001, 0.0001
**batch_size**	/	/	/	/	/	/	/	16, 32, 64	16, 32, 64	16, 32, 64
**epochs**	/	/	/	/	/	/	/	50, 100, 200	50, 100, 200	50, 100, 200
**hidden_sizes**	/	/	/	/	/	/	/	[128, 64], [256, 128]	/	/
**conv_layers**	/	/	/	/	/	/	/	/	1, 2	/
**filters**	/	/	/	/	/	/	/	/	[32, 64]	/
**kernel_size**	/	/	/	/	/	/	/	/	3, 5, 7	/
**pooling**	/	/	/	/	/	/	/	/	max_pool (2)	/
**dropout**	/	/	/	/	/	/	/	0, 0.2	0, 0.2	0, 0.2
**nhead**	/	/	/	/	/	/	/	/	/	4, 8, 16

Table S3. Top 50 TCR features ranked by permutation importance.

CDR3	Importance	TRBV	TRBJ
**CASATGASQPQHF**	0.00449294	TRBV19	TRBJ1-5
**CASGDNEQFF**	0.002310655	TRBV12-4	TRBJ2-1
**CASIHGDTEWIDYGYTF**	0.003080873	TRBV7-9	TRBJ1-2
**CASKGVSNYGYTF**	0.002481814	TRBV19	TRBJ1-2
**CASRRGTVLNEKLFF**	0.001968335	TRBV7-8	TRBJ1-4
**CASRTGQGDNSPLHF**	0.007231493	TRBV2	TRBJ1-6
**CASRTGTSDHEQFF**	0.002931108	TRBV2	TRBJ2-1
**CASSAGELFF**	0.006546855	TRBV25-1	TRBJ2-2
**CASSAGTGNYTF**	0.002139495	TRBV12-3	TRBJ1-2
**CASSASGTAYGYTF**	0.006803594	TRBV19	TRBJ1-2
**CASSFAGTSGMNEQFF**	0.002610184	TRBV7-2	TRBJ2-1
**CASSFEETQYF**	0.00885751	TRBV5-1	TRBJ2-5

**CASSFPSGARGYTF**	0.003808301	TRBV28	TRBJ1-2
**CASSFSADQETQYF**	0.002182285	TRBV5-1	TRBJ2-5
**CASSGTGSGETQYF**	0.004300385	TRBV2	TRBJ2-5
**CASSLADTQYF**	0.002567394	TRBV5-1	TRBJ2-3
**CASSLAGGTDTQYF**	0.00237484	TRBV14	TRBJ2-3
**CASSLGETQYF**	0.003102268	TRBV12-4	TRBJ2-5
**CASSLGSETQYF**	0.00415062	TRBV12-4	TRBJ2-5
**CASSLRADTQYF**	0.007744972	TRBV12-4	TRBJ2-3
**CASSLRGTHNEQFF**	0.002995293	TRBV7-6	TRBJ2-1
**CASSLTNLGGNTIYF**	0.003252033	TRBV14	TRBJ1-3
**CASSLTSGKVYEQYF**	0.002952503	TRBV5-1	TRBJ2-7
**CASSLVGYQPQHF**	0.00453573	TRBV12-4	TRBJ1-5
**CASSPERGTEAFF**	0.002866923	TRBV18	TRBJ1-1
**CASSPERSSTDTQYF**	0.005477107	TRBV9	TRBJ2-3
**CASSPGLTENYGYTF**	0.003166453	TRBV18	TRBJ1-2
**CASSPGTGGTEAFF**	0.002096705	TRBV6-1	TRBJ1-1
**CASSPNNYEQYF**	0.006675225	TRBV18	TRBJ2-7
**CASSPPSAGSNTEAFF**	0.002053915	TRBV18	TRBJ1-1
**CASSPQETQYF**	0.005926401	TRBV18	TRBJ2-5
**CASSPQPNTEAFF**	0.002396234	TRBV9	TRBJ1-1
**CASSPRDSGANVLTF**	0.001925546	TRBV11-2	TRBJ2-6
**CASSPTGGTDTQYF**	0.002439024	TRBV5-1	TRBJ2-3
**CASSQFGEETVETQYF**	0.004599914	TRBV14	TRBJ2-5
**CASSRDSNQPQHF**	0.00419341	TRBV18	TRBJ1-5
**CASSRNGGDQETQYF**	0.004557125	TRBV18	TRBJ2-5
**CASSSGSWYSYTF**	0.004706889	TRBV7-6	TRBJ1-2
**CASSSQGPSTEAFF**	0.006161746	TRBV7-3	TRBJ1-1
**CASSSRRRGVGYTF**	0.002310655	TRBV12-4	TRBJ1-2
**CASSTGTSGIPFF**	0.00419341	TRBV19	TRBJ2-1
**CASSYGRIPNQPQHF**	0.004621309	TRBV12-3	TRBJ1-5
**CASTPTSGTQETQYF**	0.001968335	TRBV7-8	TRBJ2-5
**CASTSGSDTQYF**	0.0152546	TRBV9	TRBJ2-3
**CAWSVGMNTEAFF**	0.002995293	TRBV30	TRBJ1-1
**CSAAELGHGEQYF**	0.003530167	TRBV29-1	TRBJ2-7
**CSASASGISSYNEQFF**	0.006889174	TRBV20-1	TRBJ2-1
**CSVDETGDTEAFF**	0.004043646	TRBV29-1	TRBJ1-1
**CSVLVGKVETQYF**	0.011895593	TRBV29-1	TRBJ2-5
**CSVVRLDGNEQFF**	0.002738554	TRBV29-1	TRBJ2-1

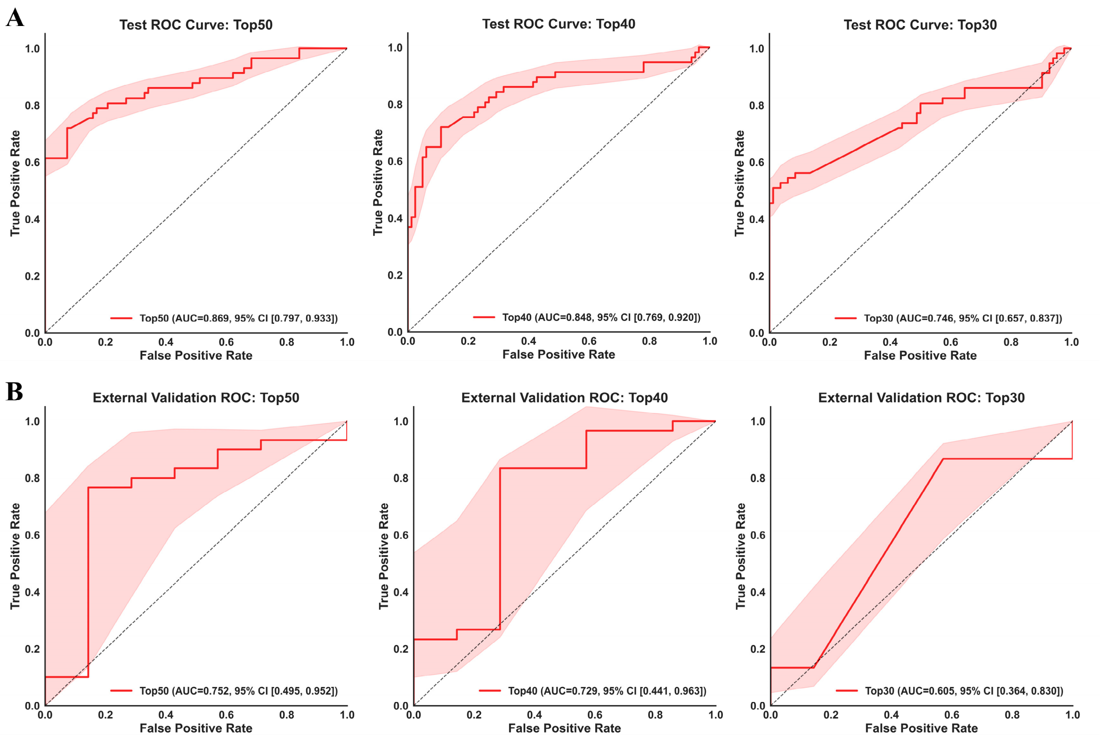

Fig. S1. Performance of Transformer models employing different numbers of features in the test cohort. (**A**) ROC curves for the internal test set with Top30, Top 40, and Top 50 features. (**B**) ROC curves for the external test set with Top30, Top 40, and Top 50 features.
